# Development and validation of a nomogram based on stromal score to predict progression‐free survival of patients with papillary thyroid carcinoma

**DOI:** 10.1002/cam4.4112

**Published:** 2021-07-09

**Authors:** Jiajia Tang, Shitao Jiang, Qiong Gao, Xuehua Xi, Luying Gao, Ruina Zhao, Xingjian Lai, Bo Zhang, Yuxin Jiang

**Affiliations:** ^1^ Department of Ultrasound Peking Union Medical College Hospital Chinese Academy of Medical Sciences and Peking Union Medical College Beijing China; ^2^ Department of Liver Surgery Peking Union Medical College Hospital Chinese Academy of Medical Sciences and Peking Union Medical College Beijing China; ^3^ Department of Medical Ultrasonics China‐Japan Friendship Hospital Beijing China

**Keywords:** nomogram, papillary thyroid carcinoma, progression‐free survival, stromal score

## Abstract

**Background:**

Growing evidence has proved that stromal cells, as the critical component of tumor microenvironment (TME), are closely associated with tumor's progression. However, the model based on stromal score to predict progression‐free survival (PFS) in papillary thyroid carcinoma (PTC) has not been developed. The study aimed at exploring the relation between stromal score and prognosis, then establishing a nomogram to predict PFS of patients with PTC.

**Method:**

We obtained the stromal score and clinicopathological characteristics of PTC patients from The Cancer Genome Atlas (TCGA) database. Cox regression analysis assisted in selecting prognosis‐related factors. A stromal score‐based nomogram was built and verified in the training and validation cohorts, respectively. The calibration curve, concordance index (C‐index), decision curve analysis (DCA) as well as receiver operating characteristic (ROC) curve assisted in measuring the performance exhibited by the nomogram.

**Results:**

We divided 381 PTC patients into the training cohort (n = 269) and the validation cohort (n = 112) randomly. Compared with patients who had a low stromal score, patients with a high stromal score appeared with significantly better PFS [Hazard ratio (*HR*) and 95% confidence interval (*CI*): 0.294, 0.130–0.664]. The C‐index of the PFS nomogram was 0.764 (0.662–0.866) in the training cohort and 0.717 (0.603–0.831) in the validation cohort. The calibration curves for PFS prediction in the nomogram were remarkably consistent with the actual observation. DCA indicated superior performance of the nomogram to predict PFS than the American Joint Committee on Cancer (AJCC) Tumor Node Metastasis (TNM) staging system. The ROC curves showed the favorable sensitivity and specificity of the novel nomogram.

**Conclusion:**

High stromal score was significantly associated with improved PFS in patients with PTC. The nomogram based on the stromal score and clinicopathological patterns yielded a reliable performance to predict the prognosis of PTC.

## Graphical abstract

High stromal score is closely associated with progression‐free survival (PFS) of patients with papillary thyroid cancer (PTC). The stromal score‐based nomogram shows superior performance to predict PFS for PTC patients than the AJCC stage system.

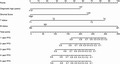

## 1 INTRODUCTION

Thyroid carcinoma acts as a common disease around the world and its incidence continues to rise in the past tens of years.^1^ Papillary thyroid carcinoma (PTC) is a representative subtype of the thyroid carcinoma. Although PTC patients manifest favorable prognosis, some patients present with aggressive progress as recurrence and metastasis, which consequently result in poor prognosis. American Joint Committee on Cancer (AJCC) Tumor Node Metastasis (TNM) staging system refers to a standard approach to predict the prognosis for PTC patients.^2^ However, it primarily focuses on death of PTC which has limitations to accurately evaluate the risk of progression in the early stage.^3^ Therefore, it is of significance to explore a novel model to predict the rate of relapse for patients with PTC.

In recent years, increasing evidence has confirmed that tumor microenvironment (TME) is closely associated with prognosis of various cancers, including PTC.^4^, ^5^ Surrounded with tumor cells, TME consists of infiltrating immune cell, stromal cell, as well as other kinds of normal epithelial cells. Abundant of studies have proved that stromal cells, as the most critical component of TME, greatly affect PTC’s progression.^6^, ^7^, ^8^ Stromal score, which could be calculated from gene expression data, was applied for estimating stromal cells’ infiltration in tumor tissue.^9^ Increasing studies have attempted to establish predictive model based on stromal score to evaluate the prognosis of tumors, such as breast cancer,^10^ gastric cancer,^11^ and clear cell renal carcinoma cancer.^12^ However, there is no report that focus on the association between stromal score and prognosis of PTC, and stromal score‐based model has not been developed to evaluate the prognosis for PTC patients.

In this study, we attempted to explore the correlation of stromal score with progression‐free survival (PFS) of PTC, and integrated stromal score with clinicopathological characteristics to build a prognostic nomogram for predicting the survival of PTC patients.

## 2 MATERIALS AND METHODS

### 2.1 Patients enrollment

The data in this study were downloaded from The Cancer Genome Atlas (TCGA) database. Clinicopathological data of PTC patients were retrieved as follows: diagnostic age, gender, race, radiation therapy status, histological subtypes, AJCC TNM stage (which is defined following the AJCC 7^th^ edition), and PFS. Detailed information is available on the following website: http://www.cbioportal.org/. Estimation of STromal and Immune cells in MAlignant Tumor tissues using Expression data (ESTIMATE) algorithm was utilized for inferring the cellularity of tumor and various infiltrating normal cells in TME. The single‐sample gene set enrichment analysis (ssGSEA) assisted in calculating the stromal score, thereby predicting the levels of infiltrating stromal cells.^9^ The stromal score of each PTC patient in our study was downloaded from the following website: http://bioinformatics.mdanderson.org/estimate/.

### 2.2 Data processing

**FIGURE 1:**
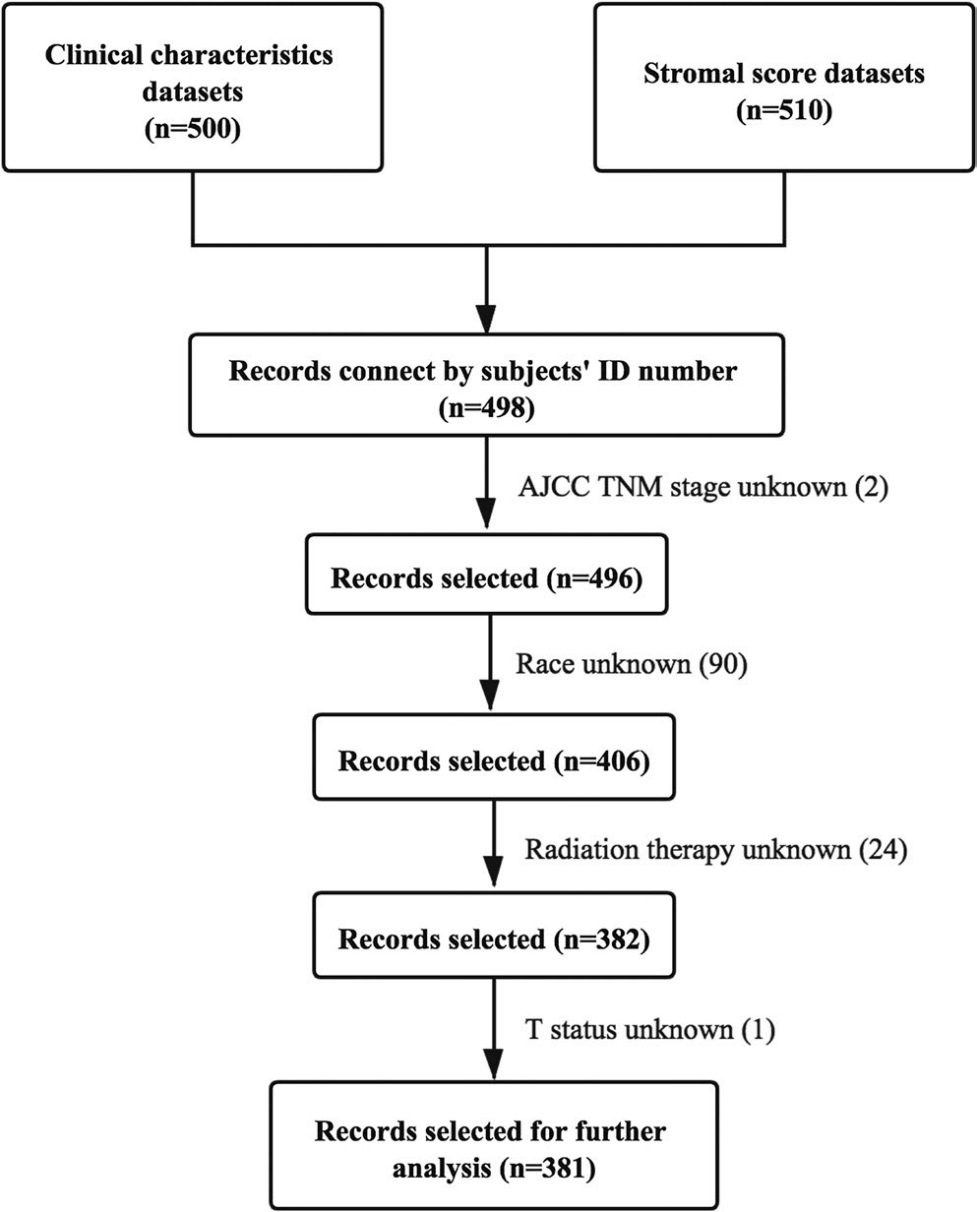
The flowchart detailing the successive steps of data processing. AJCC TNM, American Joint Committee on Cancer Tumor Node Metastasis; T, tumor

All records from the two datasets were matched by patients’ ID number. In total, 498 cases were available for screening. Some cases were excluded due to absence of information. Details of sample sizes at each stage of analysis are illustrated as a flowchart in Figure 1.

### 2.3 Development and validation of the nomogram

Poor prognosis in PTC patients is mainly caused by recurrence and metastasis, so the study was performed taking PFS as the endpoint. Using createDataPartition() function in R package, we divided people into training and validation cohorts in a 7:3 ratio (seed: 20201124). Univariate and multivariate regression models assisted in confirming the independent predictors for the PFS of PTC. We estimated the adjusted hazard ratio (*HR*) as well as the 95% confidence interval (*CI*). A nomogram was formulated using the training cohort based on the results of cox regression analyses. External validation was carried out by virtue of the validation cohort. Assessment on the performance exhibited by the nomogram was conducted through measuring the concordance index (C‐index) as well as the calibration (compare the survival probability predicted by the nomogram with the observed value by Kaplan–Meier analysis). Also, DCA assisted in confirming the threshold probability range regarding nomogram together with the AJCC TNM stage. The specificity and sensitivity of the nomogram were assessed via the receiver operating characteristics (ROC) curve.

### 2.4 Statistical analysis

Fisher's exact test or *Chi*‐square test served for the analysis of all categorized data, and Kruskal–Wallis *H* test served for the analysis of continuous variables. The optimal cutoff point was obtained by virtue of X‐tile 3.6.1 software (Yale University School of Medicine).^13^ The Kaplan–Meier method and the log‐rank test assisted in constructing and comparing the survival curves, respectively. R 3.6.3 software (http://www.r‐project.org) helped to conduct all statistical analyses. The performed statistical tests were two‐sided, with *p* values less than 0.05 were considered exhibiting a statistical significance.

## 3 RESULTS

### 3.1 The cutoff points of age and stromal score for PFS prediction

**FIGURE 2:**
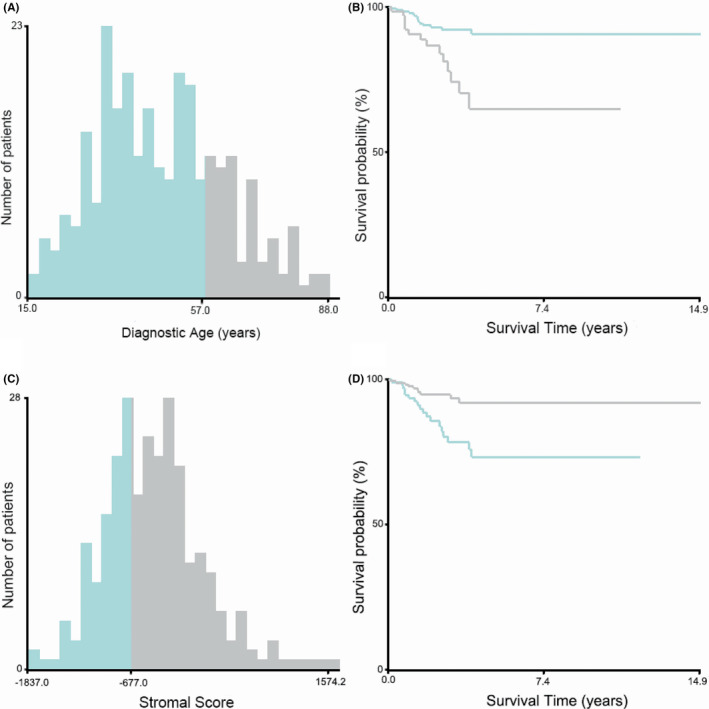
Identification of optimal cutoff points of diagnostic age and stromal score by X‐tile software analyses. (A, B) Optimal cutoff value of the diagnostic age was identified as 57 years based on progression‐free survival. (C, D) Optimal cutoff value of the stromal score was identified as −677.0 based on progression‐free survival

A total of 381 PTC patients with available data in TCGA‐THCA dataset were analyzed. The age of 57 was selected as the best cutoff point to predict PFS in PTC patients, according to the results of X‐tile plots in Figure 2A. Figure 2B showed the PFS curves specific to younger group (diagnostic age <57) and older group (diagnostic age ≥57). Patients who were younger than 57 had significantly longer PFS. Similarly, the stromal score of −677.0 was identified as the best cutoff value referring to the results of X‐tile plots in Figure 2C. Figure 2D showed the survival curves of PFS for group with a low stromal score (≤ −677.0) and group with a high stromal score (> −677.0).

### 3.2 Patients’ characteristics in training cohort and validation cohort

Table 1 listed the clinicopathological characteristics of patients analyzed in the study. In the entire dataset (n = 381), most of the patients were younger than 57 years old (74.28%), female (73.23%), White (81.63%), received radiation therapy (61.94%), and classified in AJCC TNM stage I (56.96%). Tumor status (T status), node status (N status), and metastasis status (M status) derived from AJCC TNM stage system were evaluated. Most patients belonged to T3 (35.70%), N1 (46.72%), and M0 (66.40%). Three major variants of PTC, including classical PTC (CPTC), follicular variant PTC (FVPTC) as well as tall cell PTC (TCPTC), were shown in both cohorts, and CPTC accounted for the largest proportion (75.85%). More than a half of individuals (63.52%) appeared with higher stromal scores (>−667.0). In order to evaluate the predictive model in an effective way, we divided patients in a random manner into a training cohort (n = 269) and a validation cohort (n = 112). The two cohorts did not present a significant difference.

**TABLE 1:** The clinicopathological characteristics and stromal score of PTC patients in the training and validation cohorts

Variables	Total (n = 381)	Training cohort (n = 269)	Validation cohort (n = 112)	*p*
Age (year)				0.961
≤57	283 (74.28)	200 (74.35)	83 (74.11)	
>57	98 (25.72)	69 (25.65)	29 (25.89)	
Sex				0.206
Female	279 (73.23)	192 (71.38)	87 (77.68)	
Male	102 (26.77)	77 (28.62)	25 (22.32)	
Race				0.609
White	311 (81.63)	223 (82.90)	88 (78.57)	
Black	23 (6.04)	15 (5.58)	8 (7.14)	
Other	47 (12.33)	31 (11.52)	16 (14.29)	
Radiation therapy				0.125
No	145 (38.06)	109 (40.52)	36 (32.14)	
Yes	236 (61.94)	160 (59.48)	76 (67.86)	
Histological type				0.876
CPTC	289 (75.85)	201 (74.72)	88 (78.57)	
FVPTC	54 (14.17)	39 (14.50)	15 (13.39)	
TCPTC	32 (8.41)	24 (8.92)	8 (7.14)	
Other	6 (1.57)	5 (1.86)	1 (0.90)	
AJCC TNM stage				0.450
Stage I	217 (56.96)	151 (56.13)	66 (58.93)	
Stage II	33 (8.66)	24 (8.92)	9 (8.04)	
Stage III	91 (23.88)	69 (25.65)	22 (19.64)	
Stage IV	40 (10.50)	25 (9.29)	15 (13.39)	
T status				0.669
T1	101 (26.51)	72 (26.77)	29 (25.89)	
T2	125 (32.81)	89 (33.09)	36 (32.14)	
T3	136 (35.70)	97 (36.06)	39 (34.82)	
T4	19 (4.98)	11 (4.09)	8 (7.14)	
N status				0.847
N0	173 (45.41)	124 (46.10)	49 (43.75)	
N1	178 (46.72)	125 (46.47)	53 (47.32)	
NX	30 (7.87)	20 (7.43)	10 (8.93)	
M status				0.266
M0	253 (66.40)	185 (68.77)	68 (60.71)	
M1	7 (1.84)	4 (1.49)	3 (2.68)	
MX	121 (31.76)	80 (29.74)	41 (36.61)	
Stromal score				0.974
≤ −667.0	139 (36.48)	98 (36.43)	41 (36.61)	
>−667.0	242 (63.52)	171 (63.57)	71 (63.39)	

Abbreviations: AJCC TNM, American Joint Committee on Cancer Tumor Node Metastasis; CPTC, classical PTC; FVPTC, follicular variant PTC; M, Metastasis; MX, metastasis cannot be measured; N, Node; NX, cancer in nearby lymph nodes cannot be measured; PTC, papillary thyroid carcinoma; T, Tumor; TCPTC, tall cell PTC.

### 3.3 Association of stromal score with clinicopathological characteristics and PFS in PTC patients

**FIGURE 3:**
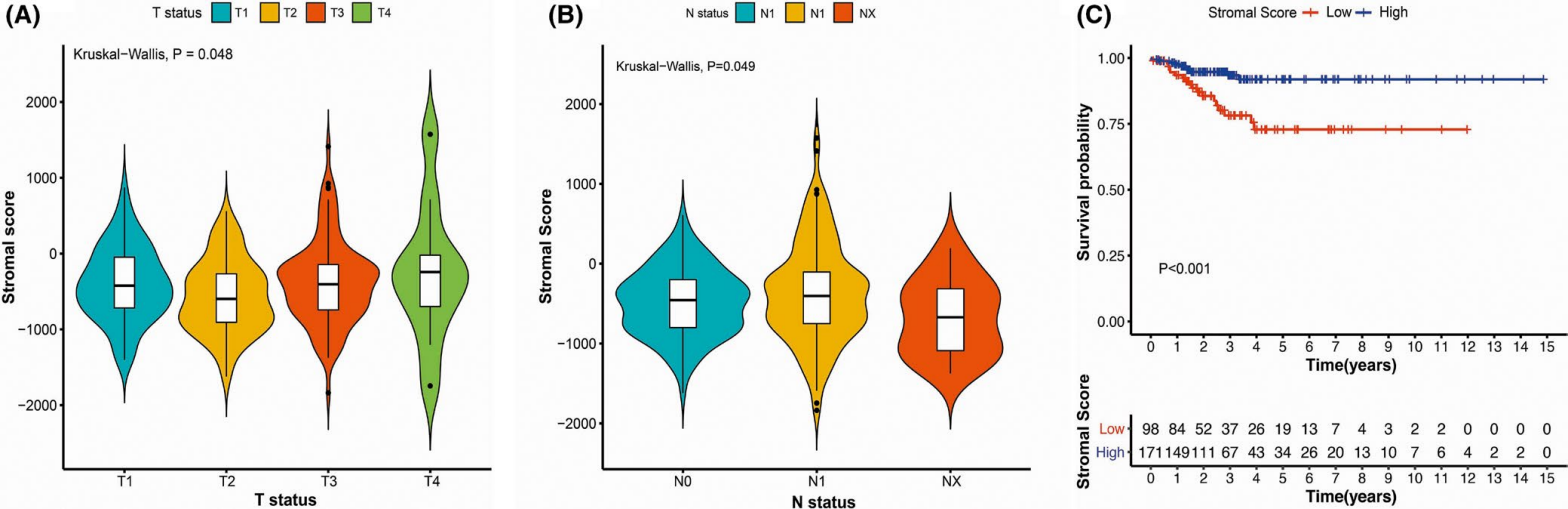
The correlations of stromal score with clinicopathological characteristics and progression‐free survival (PFS) of papillary thyroid carcinoma. (A) Correlation of the stromal score with T status. (B) Association of the stromal score with N status. (C) Comparison of PFS in patients with high and low stromal score groups. T, tumor; N, Node; NX, cancer in nearby lymph nodes cannot be measured

To explore how the stromal score related to other clinicopathological features, we divided those in the training cohort (n = 269) into two groups, namely group with a high stromal score (> −677.0) and group with a low stromal score (≤ −677.0). There were statistical differences of stromal scores among patients with different T stages (Kruskal–Wallis *H* test, *p* = 0.048)(Figure 3A). As shown in Figure 3B, patients with lymph node metastasis (N1) yielded notably lower stromal score than those without lymph node metastasis (N0) (Kruskal–Wallis *H* test, *p* = 0.049). Figure 3C displayed the association between stromal score and PFS. Patients whose stromal score was lower manifested statistically decreased PFS relative to patients whose stromal score was high (log‐rank test, *p* < 0.001).

### 3.4 The results of univariate and multivariate cox regression analyses

The univariate cox regression analysis results were shown in Table 2. There were significant differences of PFS between patients with low and high stromal scores (*p* = 0.002). It showed patients whose stromal score was higher exhibited a longer PFS (*HR* and 95%*CI*: 0.302, 0.140–0.655, *p* = 0.002). In addition, older than 57 years old were statistically associated with poorer PFS (*HR* and 95% *CI*: 3.412, 1.624–7.717, *p* = 0.001). As opposed to patients in AJCC TNM stage I, patients in stage IV were statistically associated with poorer PFS (*HR* and 95%*CI* of stage IV: 4.784, 1.313–9.117, *p* = 0.001). As expected, when compared with patients in T1, patients in T3 and T4 status had significantly poorer PFS (T3 *HR* and 95%*CI*: 5.474, 1.251–23.950, *p* = 0.024)(T4 *HR* and 95%*CI*: 11.508, 2.103–62.960, *p* = 0.005). When compared with patients in M0 (without distant metastasis), patients in M1 status (with distant metastasis) showed notably poorer PFS (*HR* and 95%*CI*: 9.386, 2.139–41.179, *p* = 0.003).

**TABLE 2:** Univariate analyses of progression‐free survival among PTC patients in the training cohort

Variables	Progression‐free survival		
	*HR*	95%*CI*	*p*
Age (year)			
≤57	1.000		
>57	3.412	(1.624, 7.717)	0.001*
Stromal score			
≤−677.0	1.000		
>−677	0.302	(0.140, 0.655)	0.002*
Sex			
Female	1.000		
Male	1.530	(0.706, 3.319)	0.282
Race			
White	1.000		
Black	0.537	(0.072, 3.986)	0.543
Other	1.212	(0.362, 4.054)	0.755
Radiation therapy			
No	1.000		
Yes	1.738	(0.765, 3.947)	0.187
Histological type			
CPTC	1.000		
FVPTC	0.758	(0.226, 2.543)	0.653
TCPTC	1.777	(0.609, 5.187)	0.293
Other	<0.001	(<0.001, <0.001)	0.997
AJCC TNM stage			
Stage I	1.000		
Stage II	1.089	(0.205, 4.029)	0.912
Stage III	1.618	(1.061, 5.099)	0.301
Stage IV	4.784	(1.313, 9.117)	0.001*
T status			
T1	1.000		
T2	2.622	(0.545, 12.630)	0.229
T3	5.474	(1.251, 23.950)	0.024*
T4	11.508	(2.103, 62.960)	0.005*
N status			
N0	1.000		
N1	2.138	(0.967, 4.728)	0.061
NX	0.903	(<0.001, <0.001)	0.996
M status			
M0	1.000		
M1	9.386	(2.139, 41.179)	0.003*
MX	1.510	(0.692, 3.295)	0.301

Abbreviations: AJCC TNM, American Joint Committee on Cancer Tumor Node Metastasis; CI, confidence interval; CPTC, classical PTC; FVPTC, follicular variant PTC; HR, hazard ratio; M, Metastasis; MX, metastasis cannot be measured; N, Node; NX, cancer in nearby lymph nodes cannot be measured; PTC, papillary thyroid carcinoma; T, Tumor; TCPTC, tall cell PTC.

* *p* < 0.05.

The results of multivariate cox proportional hazard regression analyses were listed in Table 3. Patients whose stromal score was higher had significantly improved PFS (*HR* and 95%*CI*: 0.294, 0.130–0.664, *p* = 0.003). Patients older than 57 years old were statistically presented with poorer PFS (*HR* and 95% *CI*: 5.898, 1.694–20.534, *p* = 0.005). When compared with patients in T1 status, patients in T3 status appeared with significantly poorer PFS (*HR* and 95%*CI*: 6.296, 1.217–32.555, *p* = 0.028). When compared with patients in M0 status, patients in M1 classification presented with shorter PFS (*HR* and 95%*CI*: 12.743, 1.901–85.437, *p* = 0.009). These results indicated that stromal score, age, T status, and M status were independent factors of PFS for PTC patients. Regarding the rest of the clinical characteristics, significant associations were not recognized.

**TABLE 3:** Multivariate analyses of progression‐free survival among PTC patients in the training cohort

Variables	Progression‐free survival		
	*HR*	95%*CI*	*p*
Age (year)			
≤57	1.000		
>57	5.898	(1.694, 20.534)	0.005*
Stromal score			
≤−677.0	1.000		
>−677.0	0.294	(0.130, 0.664)	0.003*
AJCC TNM stage			
Stage I	1.000		
Stage II	0.254	(0.037, 1.760)	0.165
Stage III	0.331	(0.080, 1.369)	0.127
Stage IV	0.440	(0.077, 2.504)	0.355
T status			
T1	1.000		
T2	2.506	(0.464, 13.550)	0.285
T3	6.296	(1.217, 32.555)	0.028*
T4	7.349	(0.913, 59.140)	0.061
M status			
M0	1.000		
M1	12.743	(1.901, 85.437)	0.009*
MX	1.431	(0.649, 3.156)	0.374

Abbreviations: AJCC TNM, American Joint Committee on Cancer Tumor Node Metastasis; CI, confidence interval; HR, hazard ratio; M, Metastasis; MX, metastasis cannot be measured; PTC, papillary thyroid carcinoma; T, Tumor.

* *p* < 0.05.

### 3.5 Construction and validation of the novel prognostic nomogram

**FIGURE 4:**
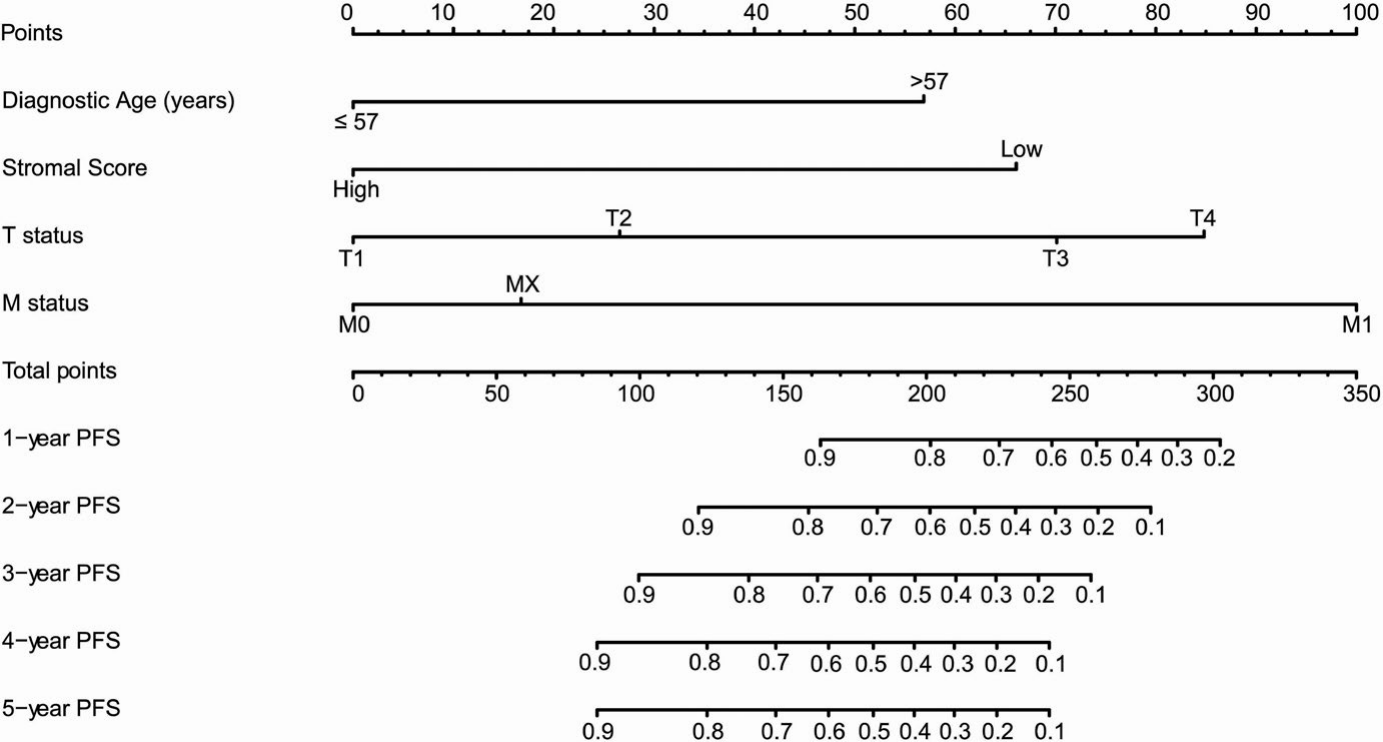
A prognostic nomogram predicting 1‐, 2‐, 3‐, 4‐, and 5‐year progression‐free survival (PFS) of papillary thyroid carcinoma. For using the nomogram, values of each variable from an individual patient are located on each variable axis, and a line is drawn upward to determine the points obtained for each variable on the point axis. The sum of these numbers is located on the total points axis, and a line is drawn downward to the survival axis to determine the likelihood of 1‐, 2‐, 3‐, 4‐, and 5‐year PFS. T, Tumor; M, Metastasis; MX, Metastasis cannot be measured

**FIGURE 5:**
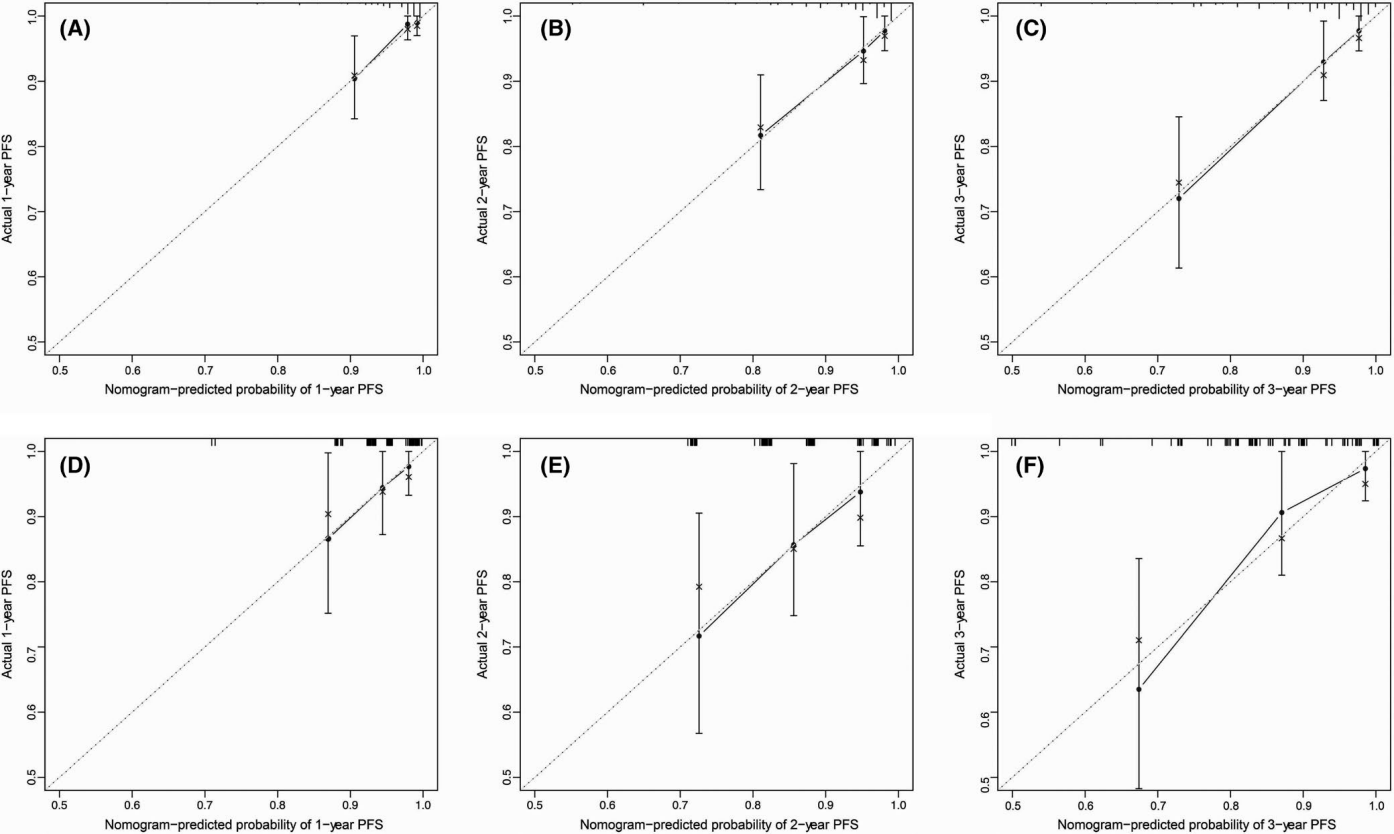
Calibration plots of progression‐free survival (PFS) associated nomogram in both training and validation cohorts. (A, B, C) Calibration plots of 1‐, 2‐, and 3‐year PFS in the training cohort. (D, E, F) Calibration plots of 1‐, 2‐, and 3‐year PFS in the validation cohort

**FIGURE 6:**
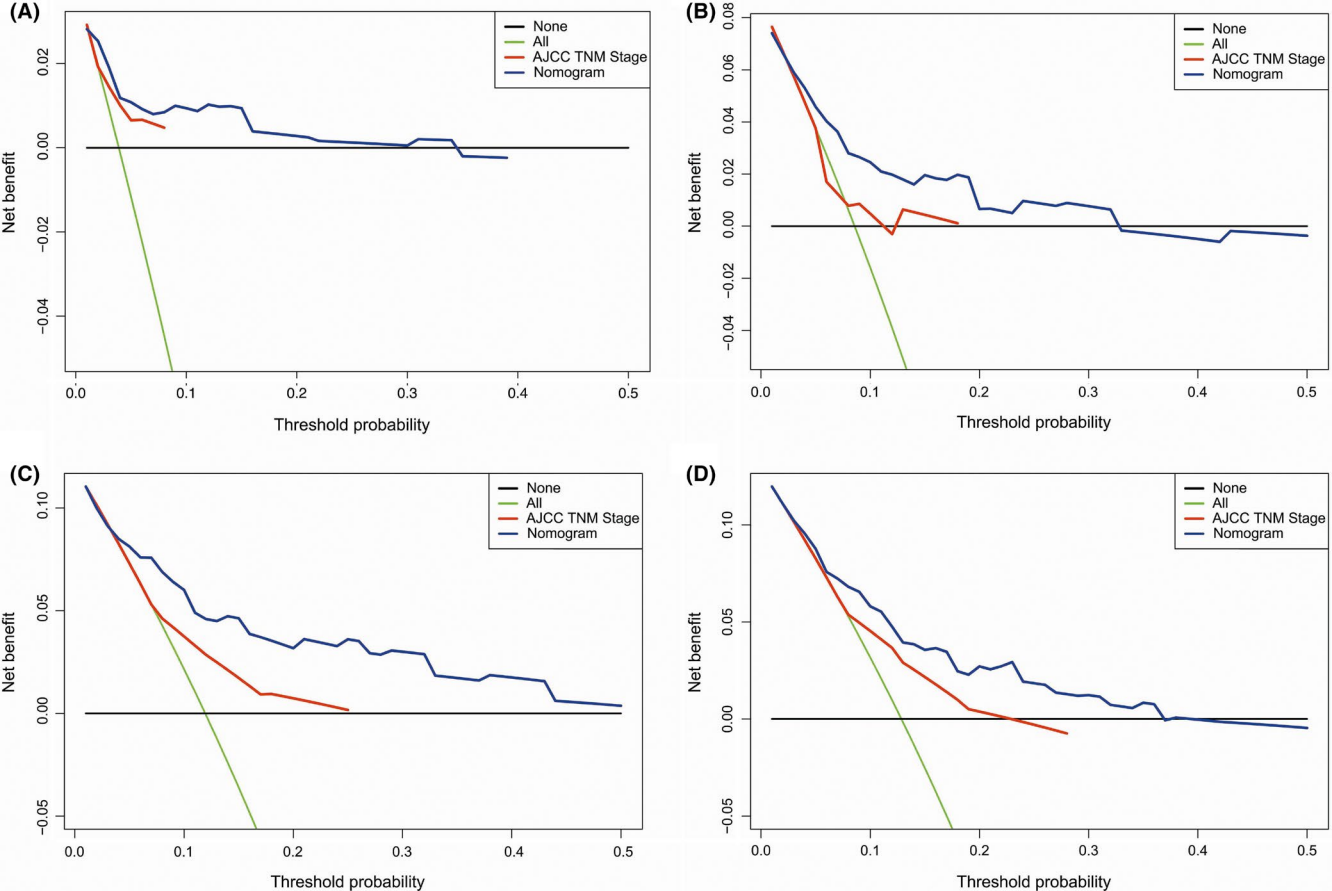
Decision curve analysis (DCA) of the nomogram for progression‐free survival (PFS) in both training and validation cohorts. (A‐C) The DCA of nomogram for predicting 1‐, 2‐, and 3‐year PFS in the training cohort. (D) The DCA of nomogram for predicting 3‐year PFS in the validation cohort. AJCC TNM, American Joint Committee on Cancer Tumor Node Metastasis

**FIGURE 7:**
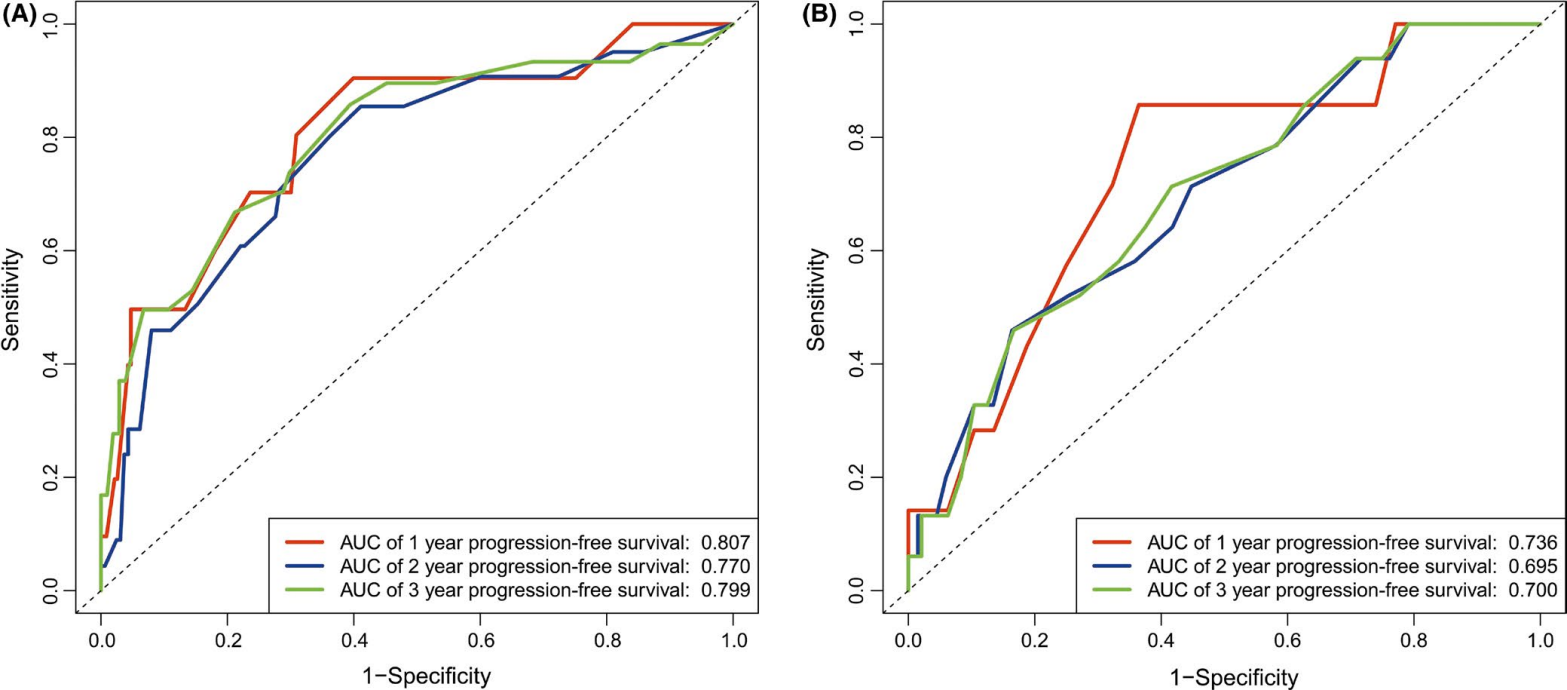
Receiver operating characteristic (ROC) curves for the nomogram. (A) The ROC curves of nomogram with 1‐, 2‐, and 3‐year progression‐free survival in the training cohort. (B) The ROC curves of nomogram with 1‐, 2‐, and 3‐year progression‐free survival in the validation cohort. AUC, area under ROC curve

Based on cox regression analyses, a nomogram was constructed for predicting PFS of PTC patients. Age, stromal score, T status, and M status were parameters included in the nomogram (Figure 4). In the training group, the C‐index of the nomogram for PFS prediction was 0.764 (95% *CI*, 0.662–0.866). Then the model was verified in the validation cohort, and the C‐index showed 0.717 (95% *CI*, 0.603–0.831). In Figure 5, as displayed in the nomogram calibration plots, the two cohorts exhibited similar predicted 1‐, 2‐, and 3‐year PFS to actual observations. The DCA results indicated that the performance exhibited by PFS nomogram was obviously better relative to the AJCC TNM stage (Figure 6). As shown in Figure 7, high area under ROC curve (AUC) showed the favorable sensitivity and specificity of the nomogram both in the training cohort (0.807, 0.770, and 0.799 for 1‐, 2‐, and 3‐year PFS, respectively), and the validation cohort (0.736, 0.695, and 0.700 for 1‐, 2‐, and 3‐year PFS, respectively). Above results indicated that the nomogram yielded reliable performance, and it showed superior predictive value relative to the traditional AJCC TNM staging system.

## 4 DISCUSSION

Despite with relatively good prognosis, PTC patients still have a risk of advanced disease. Recognizing the high‐risk patients in the early stage is critical for practitioners to select more aggressive treatment. The AJCC staging system is considered as the standard approach to predict the prognosis of PTC patients and abundant studies have indicated its applicability in clinical practice.^14^, ^15^, ^16^ However, it has limitations to identify patients with progression in the early stage, especially for the low risk majority.^3^, ^17^ The present study revealed the tight correlation between stromal score and PFS of PTC patients. Stromal score was also found to correlate with tumor status and lymph node metastasis of PTC. Based on the stromal score and other prognosis‐related patterns, we further constructed a nomogram to estimate PFS of PTC patients and it yielded a superior performance than the AJCC staging system.

Increasing research has confirmed the indispensable role of tumor microenvironment (TME) in tumor's growth and progression.^18^ As for one of the most important components of TME, stromal cells are suggested to draw critical impact on progression of various tumors.^19^, ^20^ However, the role of stromal cells in PTC has not be fully explored. ESTIMATE algorithm provides an easy way to predict immune cell and stromal cell infiltration in TME. In our study, we found the patients with higher stromal scores appeared with longer PFS, which indicate the potential role of stromal cells to prevent PTC’s progression. One previous study showed that, through secreting extracellular superoxide dismutase, stromal cells can have an inhibitory effect on thyroid cancer cell migration.^8^ Conversely, Koperek et al. demonstrated that continuous tumor growth demanded for continuous stroma expansion.^7^ They stated that the development of stroma was associated with the progression of carcinogenesis, such as lymph node metastasis, signifying that stroma responds to the microenvironmental needs of tumor cells. In addition, Liu et held that some chemotactic factor derived from stromal cells, such as SDF‐1, remarkably affect the invasion and metastasis processes of tumor cells of PTC.^6^ Overall, until now, the role of stromal cells in PTC remains unclear. Our preliminary observation could provide a perspective to explore this issue, and further research is needed in the future.

In our study, clinicopathological characteristics were found to be correlated with PFS in PTC. We defined 57 year old as the cutoff value of age, which is basically consistent with previous reports. And patients older than 57 year old showed statistically poorer PFS in our findings. Many studies have recognized 55 year old as the best single time point for prognosis model.^21^, ^22^, ^23^ And one multi‐center research demonstrated that using 55 year old as the cutoff value to predict the survival of PTC can help to avoid nearly 12% over treatment.^24^ We also found patients with distant metastasis (M1 status) presented with poorer survival, which was consistent with previous findings.^25^ Concerning with T status, patients in T3 showed significantly poorer PFS when comparing with patients in T1 status. The notable decreased PFS of patients in T3 status probably result from the extent of extrathyroidal extension (ETE).^16^ It indicated that patients with microscopic ETE were more likely to have lymph node metastases, which took a significantly higher risk of recurrence than patients without ETE.^15^, ^26^ In terms of lymph node metastasis, we found N status was not an independent factor for PFS in PTC. Some previous studies held the similar viewpoints that nodal metastasis was merely correlated with increased recurrence risk but slightly affected patients’ survival.^15^ Conversely, other researches, such as American Thyroid Association Management (ATA) Guidelines, which insisted the prognostic significance exhibited by the nodal metastasis, could be classified given the number, size, as well as extranodal invasion of the metastatic lymph nodes.^3^, ^27^ Inadequate detailed information about lymph node in TCGA database might be the limitation to show the notable association between the N status and PFS of PTC patients in our study.

In recent years, nomograms have been widely applied to estimate clinical prognosis as they integrate multiple prognostic parameters into an intuitive figure, and easier for patients to understand. Mounting studies have established nomograms considering the immune and stromal scores, aiming for taking the TME‐related cells as important factors to evaluate patients’ prognosis.^12^, ^28^ Nomograms were built to improve the prediction of prognosis in PTC patients have emerged,^17^, ^25^, ^29^, ^30^ few of which, however, took stromal scores into account. As we all know, the study for the first time constructed a prognostic model which combined stromal scores and the clinicopathological characteristics comprehensively. The new information included in our nomogram can provide novel insights in PTC’s prognosis and further assist physicians to make more effective clinical decisions.

Our study still had three major limitations. At first, we obtained the clinicopathological information for the dataset in the study mainly from the TCGA database. Most patients came from North America. Therefore, it is necessary to be cautious about applying the results of the study to patients in other places. Second, some critical prognostic factors, such as surgical treatment, multifocality, radioactive iodine, BRAF mutation, and TERT mutation, were unavailable in the TCGA database. Third, this study included the relatively small sample (n = 381). More data need to be analyzed to improve the accuracy of model performance assessments, and an external validation of the prognostic model is necessary in the further study.

## 5 CONCLUSIONS

In our study, we found PTC patients with high stromal scores were closely related to the improved PFS. We established a prognostic nomogram combining stromal score with clinicopathological parameters related to the prognosis for predicting PTC patients’ PFS. The novel nomogram showed reliable performance and could contribute to the individualized treatment as well as medical decision making.

## CONFLICT OF INTEREST

The authors declare that they have no conflict of interest.

## AUTHOR CONTRIBUTION

Jiajia Tang and Shitao Jiang collected the data and wrote the manuscript. Qiong Gao and Xuehua Xi conducted the statistical analysis. Luying Gao, Ruina Zhao, and Xingjian Lai reviewed and modified the manuscript. Bo Zhang and Yuxin Jiang approved the version to be published.

## ETHICAL STATEMENT

All information in this study was retrieved from publicly available database. Ethical review and approval were not required for the study on human participants according to the local legislation and institutional requirements.

## Data Availability

The data that support the findings of this study are openly available in The Cancer Genome Atlas Program at http://cancergenome.nih. gov/.
